# Synchronous lung adenocarcinoma *in situ* and type B2-B3 thymoma in a frozen hemithorax: a case report and multidisciplinary approach

**DOI:** 10.3389/fonc.2026.1846848

**Published:** 2026-05-15

**Authors:** Chenglin Fan, Gaoyuan Sang, Ziyi Liang, Jialin Yu, Sixuan Song, Jingjun Zhu, Zhengzhe Xu

**Affiliations:** Department of Thoracic Surgery, The Affiliated Hospital of Yanbian University (Yanbian Hospital), Yanji, China

**Keywords:** 18F-FDG PET/CT, lung adenocarcinoma *in situ*, multidisciplinary approach, synchronous double primary tumors, thymoma, uniportal VATS

## Abstract

**Background:**

Synchronous primary lung cancer and mediastinal thymoma represent a rare clinical entity. While 18F-FDG PET/CT serves as a cornerstone modality for differentiating benign from malignant thoracic neoplasms, hypermetabolic activity secondary to severe localized inflammation can frequently masquerade as highly aggressive malignancies. Such radiologic-pathologic discordance significantly elevates the risk of misdiagnosis, thereby adding substantial complexity to clinical decision-making and patient management.

**Case presentation:**

A 73-year-old woman with prior tuberculosis and stented coronary artery disease presented with a hypermetabolic right upper lobe (RUL) nodule (2.3 cm, SUVmax 5.0) and an anterior mediastinal mass (SUVmax 3.0) on PET/CT. Due to the patient’s cardiovascular comorbidities, the multidisciplinary team (MDT) initially recommended a percutaneous biopsy. As the pathology revealed only adenocarcinoma *in situ* (AIS) in the context of a persistent cough and a hypermetabolic lesion, empirical broad-spectrum antibiotics were administered. A two-month follow-up HRCT showed substantial shrinkage of the right lung lesion and absorption of its solid component. The patient subsequently underwent elective uniportal video-assisted thoracoscopic surgery (VATS) for a right upper lobe posterior segmentectomy and an anterior mediastinal tumor resection. Intraoperatively, dense pan-pleural adhesions from the prior tuberculosis were evident, compounded by localized adhesions from the recent biopsy. Following sharp adhesiolysis, both lesions were successfully resected. Final pathology revealed a diagnostic masquerade: the true AIS measured only 0.66 cm, obscured by surrounding organizing pneumonia, occurring synchronously with a B2-B3 thymoma (Ki-67 >30%). The postoperative course was uneventful. The patient was discharged on postoperative day 6 after chest tube removal, and is scheduled for regular radiological follow-up.

**Conclusion:**

This report highlights a critical diagnostic pitfall: the “hypermetabolic inflammatory trap” on PET/CT can easily mimic or obscure true micro-carcinomas. In medically high-risk cohorts, leveraging an MDT approach with upfront tissue diagnosis, diagnostic anti-infective management, and dynamic imaging is paramount to avoiding surgical overtreatment. Crucially, we validate that despite the technical hurdles of extensive pleural adhesions, uniportal VATS remains a highly secure and advantageous strategy for resecting synchronous thoracic neoplasms.

## 1 Introduction

Synchronous multiple primary thoracic tumors (SMPT), such as concurrent primary lung cancer and mediastinal neoplasms, are clinically rare entities. Although their detection rates have improved owing to the widespread application of HRCT and 18F-FDG PET/CT, they remain a formidable diagnostic and therapeutic challenge (1). This is particularly evident when micro-carcinomas are surrounded by severe inflammation, organizing pneumonia, or hemorrhage; such lesions can easily masquerade as highly aggressive malignancies due to moderately elevated FDG avidity, resulting in a striking radiologic-pathologic discordance (2). When such complex dual primary tumors manifest in an elderly patient with a high cardiovascular risk profile (requiring chronic anticoagulation) and prior tuberculosis, the surgical morbidity is exceedingly high. Herein, we report a rare, high-risk case of SMPT: complicated by false-positive hypermetabolism on PET/CT secondary to severe inflammation. Following dynamic evaluation by a multidisciplinary team (MDT), our surgical team successfully performed a synchronous resection via uniportal video-assisted thoracoscopic surgery (VATS), navigating the extreme dense adhesions of a severe “frozen hemithorax.”

## 2 Case description

### 2.1 Patient information

A 73-year-old female non-smoker was admitted following the incidental finding of a right upper lobe nodule and a mediastinal mass during a routine physical exam two months prior. She reported a recent cough, but denied hemoptysis, fever, chest pain, or dyspnea. Her medical history includes hypertension, which is well-controlled on medication, and coronary artery disease treated with PCI and stenting 10 years ago; she remains on long-term antiplatelet therapy. She also had completely resolved childhood pulmonary tuberculosis, which places her at risk for severe pleural adhesions.

### 2.2 Clinical findings and imaging evaluation

In January 2026, a PET/CT scan revealed a 2.3 × 1.7 cm soft-tissue nodule in the posterior segment of the right upper lobe. The lesion demonstrated spiculation, pleural indentation, internal bronchial distortion, peripheral cavitation, and elevated 18F-FDG uptake (SUVmax 5.0) (**Figures 1A–C**). Although these fibrotic and hypermetabolic features strongly suggested an aggressive peripheral lung cancer (3), the cavitation and the patient’s history of tuberculosis necessitated a differential diagnosis to rule out severe organizing pneumonia or specific infections. Concurrently, an oval soft-tissue mass (3.4 cm × 1.9 cm) was identified in the anterior mediastinum, demonstrating mildly heterogeneous FDG avidity (SUVmax 3.0), which favored a benign or low-grade malignant neoplasm (**Figure 1D**).

**Figure 1:**
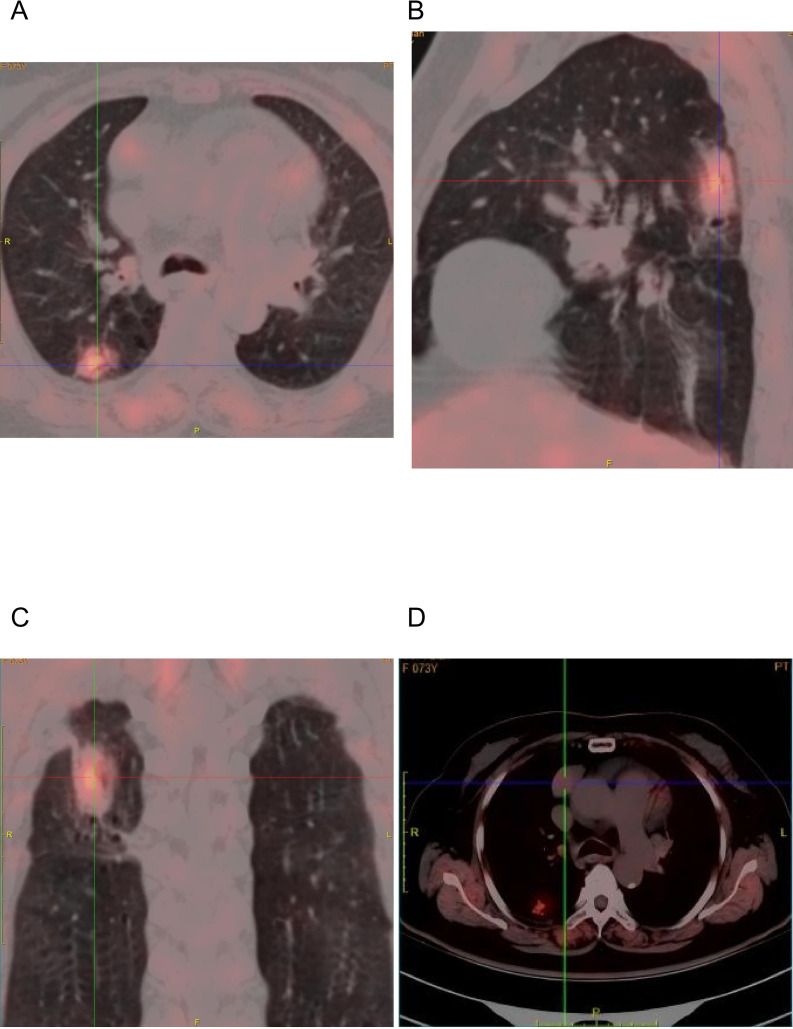
Initial 18F-FDG PET/CT findings. **(A–C)** Coronal and sagittal multiplanar reconstructions (MPR) and PET images of the right upper lobe nodule, demonstrating high FDG uptake (SUVmax 5.0) with marginal spiculation, pleural indentation, and focal cavitation. **(D)** Axial fused PET/CT image showing a mildly hypermetabolic anterior mediastinal mass.

### 2.3 MDT decision-making and surgical intervention

Given the patient’s advanced age and exceedingly high cardiovascular risk profile, the MDT elected to prioritize an upfront CT-guided percutaneous right lung biopsy, which histopathologically confirmed adenocarcinoma *in situ* (AIS) (**Figure 2**). Suspecting a secondary infection due to her cough and the lesion’s hypermetabolism, we administered a 10-day course of piperacillin-tazobactam (4.5 g every 12 hours). This regimen was specifically selected to minimize hepato-renal risks given her advanced age. A follow-up high-resolution CT (HRCT) in mid-March 2026 demonstrated significant regression of the right upper lobe nodule, characterized by the marked absorption of its solid components (**Figures 3A–C**). The integration of these dynamic radiological changes with the biopsy findings strongly suggested that the intense hypermetabolism on the initial PET/CT was, in fact, caused by severe peritumoral inflammation masking the true micro-carcinoma.

**Figure 2:**
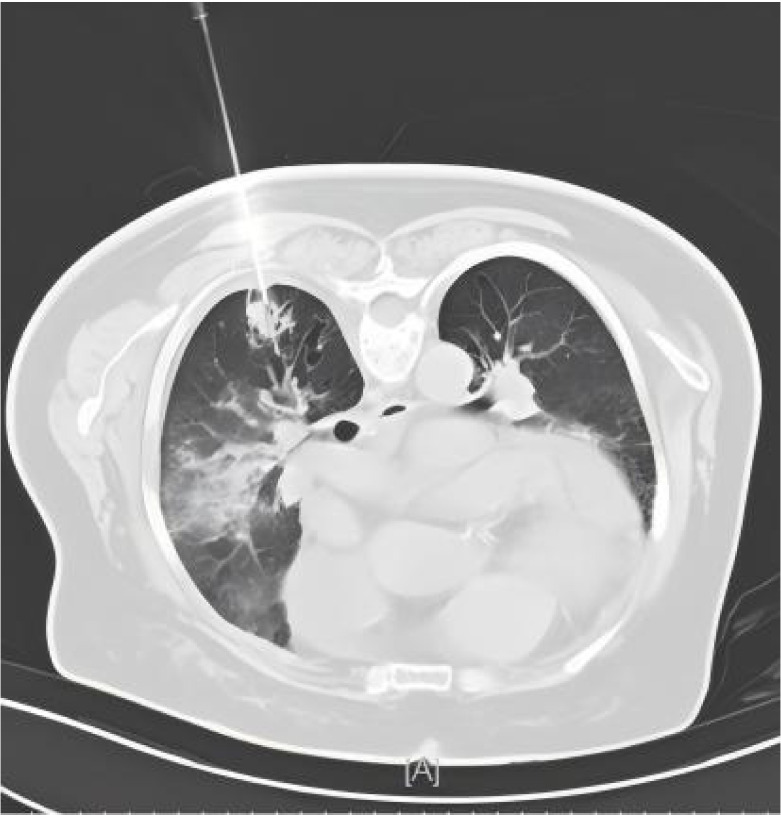
CT-guided percutaneous needle biopsy. Axial CT image showing the biopsy needle accurately positioned within the hypermetabolic lesion in the right upper lobe. The pathological result later confirmed a microscopic adenocarcinoma *in situ* (AIS).

**Figure 3:**
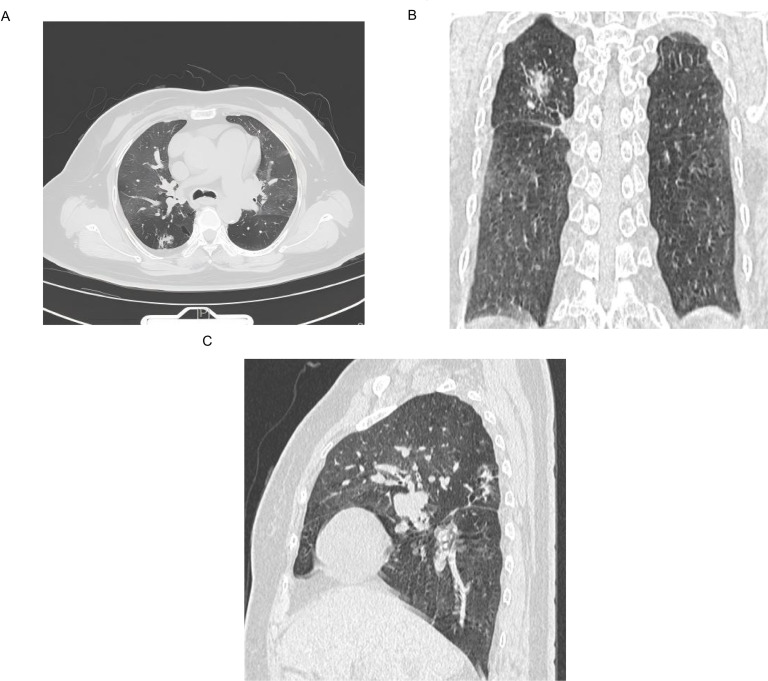
Post-treatment HRCT findings. High-resolution CT images obtained 2 months after empirical piperacillin-tazobactam therapy. **(A–C)** Axial, coronal, and sagittal views show marked shrinkage of the right upper lobe nodule and significant absorption of its solid component.

In light of the concurrent anterior mediastinal mass, an elective synchronous minimally invasive resection via uniportal VATS was scheduled (4). The right hemithorax was entered through the fifth intercostal space. Intraoperative exploration revealed extensive, dense pleural adhesions from prior tuberculosis, which were particularly severe immediately surrounding the lesion due to the recent needle biopsy. Following adhesiolysis, the surgical team successfully executed a wedge resection of the right upper lobe posterior segment nodule alongside an extended resection of the anterior mediastinal lesion.

### 2.4 Pathological diagnosis and staging

Pathology of the right lung lesion (0.66 × 0.33 cm) revealed adenocarcinoma *in situ* (AIS) surrounded by organizing pneumonia, which clinically correlates with the initial false-positive hypermetabolism (**Figure 4A**) (5). The anterior mediastinal mass was an encapsulated type B2-B3 thymoma with a high Ki-67 index (>30%) (**Figures 4B, C**) (1). The final pathological staging was pTisN0M0 (Stage 0) for the lung AIS (6), and Masaoka-Koga Stage I (7, 8)/pT1aN0M0 (Stage I, AJCC 8th ed.) (9) for the thymoma.

**Figure 4:**
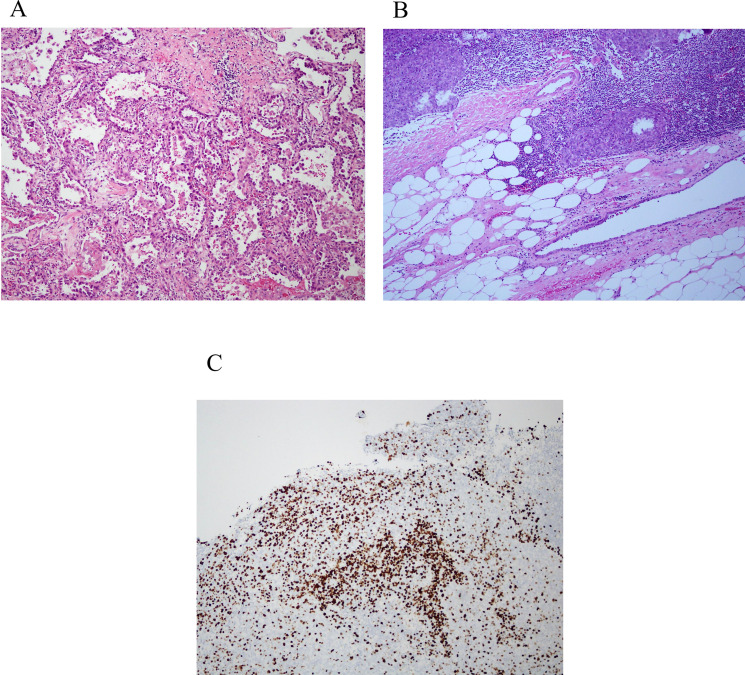
Postoperative histopathology and immunohistochemistry. **(A)** H&E staining of the right lung lesion shows adenocarcinoma *in situ* (AIS), with neoplastic cells proliferating along alveolar septa without stromal invasion. **(B)** H&E staining of the anterior mediastinal mass reveals a type B3 thymoma, composed of sheet-like epithelial tumor cells and sparse non-neoplastic lymphocytes. **(C)** Ki-67 staining of the thymoma indicates a high proliferation index (>30%).

### 2.5 Postoperative course and follow-up

Given the extensive raw surface area resulting from widespread intraoperative adhesiolysis, a single 22-French (22F) chest tube was routinely placed. The patient’s postoperative course was uneventful, with stable vital signs throughout. By postoperative day 6, the pleural drainage met the standard criteria for removal (< 150 mL/d with no evidence of air leak or active hemorrhage); consequently, the chest tube was extracted, and she was safely discharged. In light of her exceptionally high-risk cardiovascular comorbidities, the MDT consensus was to omit adjuvant radiotherapy for the resected thymoma, opting instead for strict radiographic surveillance (10) (**Figure 5**).

**Figure 5:**
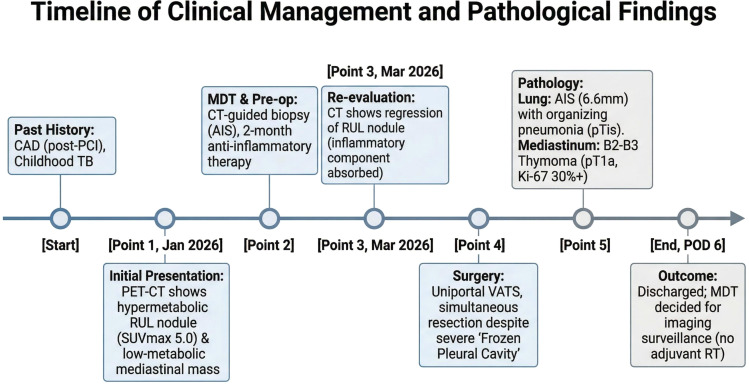
Timeline of clinical management and pathological findings.

## 3 Discussion

### 3.1 Radiologic-pathologic discordance: mechanisms of inflammation-induced hypermetabolism

Initially, the lesion exceeded 2 cm in diameter with an SUVmax of 5.0. Its imaging features—spiculation, pleural indentation, and bronchial distortion—strongly mimicked an aggressive peripheral lung cancer. However, the lesion shrank significantly following standard antimicrobial therapy. Final surgical pathology revealed a minute AIS surrounded by extensive chronic inflammation and organizing pneumonia, indicating that the initial hypermetabolism was primarily driven by a localized inflammatory response.

The false-positive PET-CT findings stem from overlapping metabolic mechanisms. While neoplastic 18F-FDG uptake is driven by aerobic glycolysis and glucose transporter (GLUT) overexpression, activated macrophages and neutrophils in severe inflammation similarly upregulate GLUTs and hexokinases to fuel phagocytosis and the respiratory burst. This shared metabolic profile explains the radiologic-pathologic discordance observed here (11).

The diagnosis of OP was made retrospectively from the surgical specimen; the initial needle biopsy, limited by sampling error, had shown only AIS. Given the patient’s cough and the lesion’s hypermetabolism, empirical broad-spectrum antibiotics were administered. Because OP generally responds to corticosteroids rather than antibiotics (12), the marked regression of the solid component on antimicrobials alone suggests the initial high FDG uptake was not exclusively due to OP. Acute and chronic bacterial inflammation at the tumor periphery likely contributed to this false-positive hypermetabolic appearance. Ultimately, this case underscores the value of multidisciplinary team (MDT) management: combining diagnostic antimicrobial therapy with dynamic imaging follow-up can effectively differentiate inflammatory mimics from true malignancy, preventing overly aggressive surgical resections.

### 3.2 Uncoupling of proliferative activity and metabolism: an analysis of the Ki-67/SUVmax mismatch

Typically, a positive correlation exists between a tumor’s FDG uptake (SUVmax) and its cellular proliferation index (Ki-67) (13). However, the type B2-B3 thymoma in the present case exhibited a paradoxical phenomenon characterized by “high proliferation (Ki-67 > 30%) and low metabolism (SUVmax 3.0)” (14). A review of the literature indicates that such mismatches in thymic epithelial tumors (TETs) are primarily attributable to the following mechanisms:

First, a metabolic dilution effect. This subtype of thymoma is abundantly infiltrated by non-neoplastic lymphocytes; while their active proliferation elevates the overall Ki-67 labeling index, their inherently low basal glucose metabolic rate effectively dilutes the average FDG avidity of the entire lesion (14). Second, attenuated glycolytic capacity. Compared to thymic carcinomas, these neoplastic cells often exhibit lower membrane expression of glucose transporters (e.g., GLUT1), resulting in a glucose uptake capacity that falls short of their high proliferative potential (15). Third, microenvironmental constraints. Inadequate tumor neoangiogenesis or a high stromal ratio can mechanically impede FDG delivery to the tumor bed (16).

This paradoxical presentation robustly demonstrates that when evaluating anterior mediastinal masses, clinicians must not subjectively rule out high proliferative potential based solely on “hypometabolic” PET/CT features. Definitive characterization must strictly adhere to the gold standard of histopathology and immunohistochemistry.

### 3.3 Synchronous multiple primary tumors with severe pleural adhesions: MDT management and surgical strategy

Managing synchronous thoracic tumors in this patient involved navigating severe, dense pleural adhesions caused by her prior tuberculosis, which were further exacerbated by exudation from the recent preoperative biopsy. These adhesions obscured anatomical landmarks and prevented complete lung deflation, severely limiting surgical exposure. Consequently, sharp dissection carried a substantial risk of massive hemorrhage from avulsed major vessels, such as pulmonary artery branches, or intractable air leaks from parenchymal injury (17).

A staged surgical approach was ruled out. Subjecting an elderly patient with stented coronary artery disease to a second general anesthesia and repeated interruptions of her antithrombotic regimen would carry unacceptable cardiovascular and cerebrovascular risks. Instead, we performed a single-stage complete sharp adhesiolysis and radical resection of both lesions via uniportal VATS (18).

Postoperatively, although the resected thymoma exhibited a high proliferative index (Ki-67 >30%), the MDT recommended close surveillance rather than adjuvant radiotherapy. This decision was deliberately tailored to preserve her limited cardiovascular reserve and prevent severe cardiopulmonary toxicities.

### 3.4 Diagnostic challenges and future directions

This report outlines an MDT-guided approach for managing synchronous primary thoracic tumors in a high-risk patient, demonstrating the clinical utility of a step-wise pathway: pathologic diagnosis, followed by diagnostic antimicrobial therapy, and ultimately, minimally invasive synchronous resection.

The case also highlights the diagnostic limits of standard 18F-FDG PET/CT, particularly its inability to reliably distinguish minute AIS from severe localized inflammation. Currently, differentiating the two relies on invasive biopsies or prolonged therapeutic trials with serial imaging—approaches that inevitably increase both healthcare costs and patient anxiety.

Improving non-invasive diagnostic tools is essential to address this clinical gap. Advanced radiomics could help quantify textural differences between inflammatory and malignant lesions (19). Concurrently, novel tumor microenvironment-targeted radiotracers, such as FAPI (20), and highly sensitive liquid biopsies, including ctDNA methylation assays (21), offer alternative diagnostic avenues. Integrating these modalities will ultimately provide a more robust evidence base for managing indeterminate, high-risk pulmonary nodules.

### 3.5 Study limitations

This report is inherently limited by its nature as a single case study; therefore, the broader applicability of this clinical pathway requires validation in larger cohorts. Additionally, because the patient’s poor performance status precluded adjuvant radiotherapy for the aggressive thymoma, prolonged follow-up is necessary to accurately evaluate her long-term survival outcomes.

## 4 Conclusion

The coexistence of minute AIS and severe localized inflammation can result in false-positive hypermetabolism on PET/CT. In high-risk patients with cardiovascular comorbidities and dense pleural adhesions, extensive surgical resection based solely on suspected aggressive imaging features should be avoided. A step-wise multidisciplinary approach—combining biopsy, diagnostic antimicrobial therapy, and serial imaging—can effectively clarify the diagnosis. Additionally, single-stage uniportal VATS remains a viable and safe option for synchronous tumor resection, even in the presence of severe thoracic adhesions. This strategy minimizes the risk of overtreatment while maximizing perioperative safety for vulnerable populations.

## Data Availability

The original contributions presented in the study are included in the article/supplementary material. Further inquiries can be directed to the corresponding author.
